# Characteristics of Quinolone Resistance in *Salmonella* spp. Isolates from the Food Chain in Brazil

**DOI:** 10.3389/fmicb.2017.00299

**Published:** 2017-03-14

**Authors:** Bruno R. Pribul, Marcia L. Festivo, Marcelle S. Rodrigues, Renata G. Costa, Elizabeth C. dos P. Rodrigues, Miliane M. S. de Souza, Dalia dos P. Rodrigues

**Affiliations:** ^1^National Reference Laboratory for Enteric Diseases, Oswaldo Cruz Institute(FIOCRUZ)Rio de Janeiro, Brazil; ^2^Laboratory of Veterinary Bacteriology, Federal Rural University of Rio de Janeiro, UFRRJRio de Janeiro, Brazil

**Keywords:** foodborne diseases, *Salmonella* spp., quinolone resistance, plasmid mediated quinolone resistance, clonal profile

## Abstract

*Salmonella* spp. is an important zoonotic pathogen related to foodborne diseases. Despite that quinolones/fluoroquinolones are considered a relevant therapeutic strategy against resistant isolates, the increase in antimicrobial resistance is an additional difficulty in controlling bacterial infections caused by *Salmonella* spp. Thus, the acquisition of resistance to quinolones in *Salmonella* spp. is worrisome to the scientific community along with the possibility of transmission of resistance through plasmids. This study investigated the prevalence of plasmid-mediated quinolone resistance (PMQR) in *Salmonella* spp. and its association with fluoroquinolone susceptibility in Brazil. We evaluated 129 isolates, 39 originated from food of animal sources, and 14 from environmental samples and including 9 from animals and 67 from humans, which were referred to the National Reference Laboratory of Enteric Diseases (NRLEB/IOC/RJ) between 2009 and 2013. These samples showed a profile of resistance for the tested quinolones/fluoroquinolones. A total of 33 serotypes were identified; *S*. Typhimurium (63) was the most prevalent followed by *S*. Enteritidis (25). The disk diffusion test showed 48.8% resistance to enrofloxacin, 42.6% to ciprofloxacin, 39.53% to ofloxacin, and 30.2% to levofloxacin. According to the broth microdilution test, the resistance percentages were: 96.1% to nalidixic acid, 64.3% to enrofloxacin, 56.6% to ciprofloxacin, 34.1% to ofloxacin, and 30.2% to levofloxacin. *Qnr* genes were found in 15 isolates (8 *qnr*S, 6 *qnr*B, and 1 *qnr*D), and the aac(6′)-Ib gene in 23. The integron gene was detected in 67 isolates with the variable region between ±600 and 1000 bp. The increased detection of PMQR in *Salmonella* spp. is a serious problem in Public Health and must constantly be monitored. Pulsed-field gel electrophoresis was performed to evaluated clonal profile among the most prevalent serovars resistant to different classes of quinolones. A total of 33 pulsotypes of *S*. Typhimurium were identified with a low percentage of genetic similarity (≤65%). This result demonstrates the presence of high diversity in the resistant clones evaluated in this study.

## Introduction

Foodborne diseases caused by *Salmonella* spp. are a serious public health problem in many parts of the world. The variety of food sources, particularly foods of animal origin, and routes of transmission can lead to human infection (Scallan et al., 2011).

In addition, the progressive increase of antimicrobial resistance in foodborne Salmonella isolates is observed as due to the uncontrolled use of these drugs for therapeutic and prophylactic purposes in foods of animal origin such as poultry, pigs, and cattle. These events have reinforced the need for epidemiological studies describing the prevalence and patterns of resistance in these bacteria (Yang et al., 2010; Tamang et al., 2011). Antimicrobial-resistant bacteria emerge from the use of antimicrobial drugs to treat and prevent diseases and promote growth in large-scale animal production.

Quinolones, particularly fluoroquinolones, are commonly used for the treatment of multi-drug resistant salmonellosis “in human and veterinary medicine” because of their broad spectrum antimicrobial activity (Dalhoff, 2012).

Point mutations in DNA gyrase and topoisomerase IV genes are directly related to quinolone resistance in *Enterobacteriaceae* “by changes in the action target site called quinolone resistance-determining regions (QRDR).” “In *Salmonella* spp., these mutations are related to resistance to nalidixic acid (NAL) and reduced susceptibility to FQs such as that of ciprofloxacin (Cip) (Cavaco and Aarestrup, 2009).” It is believed that the resistance to quinolones is mediated only by this mechanism. However, the situation changed with the discovery of a variety of determinants of plasmid-mediated quinolone resistance (PMQR).

Currently three mechanisms are recognized as PMQRs. The *qnr* genes with five different qnr families, each with different numbers of alleles “(qnrA1–7, qnrS1–4, qnrB1–31, qnrC, and qnrD) (Jacoby et al., 2009)”; “a modified aminoglycoside acetyl-transferase gene [aac(6′)-1b-cr] (Robicsek et al., 2005)”; and a specific quinolone efflux pump (qepA) (Yamane et al., 2007) and multidrug resistance pumps such as oqxAB (Zhao et al., 2010). PMQR-positive isolates present a low-level of resistance to quinolones (only a small reduction in susceptibility to nalidixic acid). However, the ability to highlight pre-existing resistance mechanisms, such as chromosomal mutations in target regions of quinolones that still allow the selection of resistant mutants to quinolone concentrations (therapeutic doses), emphasize the importance of studying these genes (Cui et al., 2014).

The present study identified the occurrence of some PMQR in *Salmonella* spp. isolated between 2009 and 2013 from the food chain in Brazil, and characterized the genetic similarity profile of serovars of greatest importance in the dispersion of resistance to quinolones in Brazil.

## Materials and methods

### Bacterial isolates

129 *Salmonella* spp. strains with resistance to quinolone and/or fluoroquinolone were evaluated. Of these, 51.9% (67/129) were from human clinical isolates, 30.2% (39/129) from food products for human consumption (beef, eggs, milk), 7.1% (9/129) from food of animal origin for human consumption (poultry, swine, cattle), and 10.8% (14/129) from environmental samples (water and drag swabs); all samples were selected from a database. The studied strains were sent to the National Reference Laboratory of Enteric Diseases (NRLEB/IOC/RJ) between 2009 and 2013 and stored in phosphate-buffered agar at room temperature and/or in BHI/glycerol broth −70°C. The isolates were inoculated in Nutrient Broth (DIFCO) and incubated at 37°C for 12–18 h for subsequent tests, as confirmation of the biochemical, serological and antimicrobial resistance profile.

### Antigenic characterization

The serological determination of *Salmonella* serotypes was determined according to the Kauffmann-White scheme using slide agglutination with O and H antisera prepared in the LRNEB/IOC/RJ.

### Antimicrobial susceptibility

The obtained resistance profiles were confirmed by the disk diffusion test according to Clinical and Laboratory Standards Institute (2013, 2014). According to Pribul et al. (2016), this test was performed using representatives of the quinolone class (OXOID) for human and veterinary therapeutic use, such as Nalidixic Acid (NAL), Ciprofloxacin (CIP), Enrofloxacin (ENO), Ofloxacin (OFL), and Levofloxacin (LVX).

MIC determinations for Nalidixic Acid (SIGMA), Ciprofloxacin (SIGMA), Enrofloxacin (SIGMA), Levofloxacin (SIGMA), and Ofloxacin (SIGMA) were performed in 96-well-microplates and according to the Clinical and Laboratory Standards Institute (2013) broth microdilution assay.

### Detection of PMQR

Total DNA was extracted using the DNEASY Tissue Qiagen® kit. The studied genes were detected by PCR amplification using primer sequences reported in Pribul et al. (2016). The *qnrA, qnrB*, and *qnrS* genes were amplified through multiplex PCR reactions; the *rrs* gene was used as the reaction control. The *qnrC, qnrD, aac(6*′*)-Ib, integrase*, and variable *integron* region genes were amplified by simplex PCR.

### PFGE

The isolates from serovars *S*. Typhimurium, *S*. Muenchen, *S*. Infantis, and *S*. Heidelberg were subjected to molecular typing by pulsed-field gel electrophoresis, which clonally evaluates isolates. The PulseNet protocol was used in this study including DNA preparation according to Heir et al. (2000) and digestion with XbaI restriction enzyme, according to Pfaller et al. (1992), Tenover et al. (1997), and Cooper et al. (2006). The definition of clones was based on the recommendations of Tenover et al. (1997) and Barrett et al. (2006). *S*. Braenderup H9812, which is considered the universal strain for PulseNet (Hunter et al., 2005), was used as the standard. The restriction patterns were analyzed in the BioNumerics software IV (Applied Maths).

## Results

### Serovar identified

Altogether, 26 different *Salmonella* serovars were identified. *Salmonella* Typhimurium (48.8%, 63/129) was the predominant serovar followed by *Salmonella* Enteritidis (19.4%, 25/129). The prevalent serovars associated with resistance to quinolones are presented in Table 1.

**Table 1:** **Distribution of quinolone-resistant *Salmonella* spp. serovars isolated from food chain diseases**.

***Salmonella*** **spp. Serotype**	**Number of NTS^a^ from**				
	**Human**	**Food**	**Environment**	**animal**	**Total**
*S*. Typhimurium	35	22	4	2	63
*S*. Enteritidis	24	1	–	–	25
*S*. Muenchen	2	2	–	–	4
*S*. Infantis	1	1	–	–	3
*S*. Heidelberg	2	–	–	1	3
Others	3	11	8	5	26
Total	67	37	12	8	

a *Non-typhoidal Salmonella*.

Most of the studied samples were isolated in 2012 (88 of 129).

Among these 129 isolates that were previously resistant to Nalidixic Acid, five were sensitive to all tested quinolones (including Nalidixic Acid), 55 (42.6%) were resistant to Ciprofloxacin, 63 (48.8%) to Enrofloxacin, 51 (39.53%) to Ofloxacin, and 48 (37.2%) to Levofloxacin in the disc diffusion test.

The broth microdilution test identified 36.4% (47/129) isolates with decreased susceptibility to Ciprofloxacin (MICs between 0.125 and 0.5 mg/ml), 20.1% (26/129) to Enrofloxacin, 9.3% (12/129) to Ofloxacin, and 6.2% (8/129) to Levofloxacin (MICs between 0.5 and 1 mg/ml). The decreased susceptibility breakpoint to Nalidixic Acid is not reported by Clinical and Laboratory Standards Institute (2015). Seventy-three (56.6%) isolates were resistant to Ciprofloxacin, 83 (64.3%) to Enrofloxacin, 44 (34.1%) to Ofloxacin, and 39 (30.2%) to Levofloxacin. A total of 124 isolates (96.1%) were resistant to Nalidixic Acid.

The resistance profile obtained with the microdilution test showed that 37 (28.7%) isolates were resistant to all tested quinolones, 30 (23.2%) to Ciprofloxacin, Enrofloxacin, and Nalidixic Acid, 16 (12.4%) to Enrofloxacin and Nalidixic Acid, 2 (1.5%) to Ciprofloxacin and Nalidixic Acid, and 39 (30.2%) to Nalidixic Acid only.

The detection of resistance genes showed six isolates carrying the *qnr*B gene, eight the *qnr*S gene, and one the *qnr*D gene. Among these 15 positive isolates, 10 strains were recovered from human samples, 3 from food of animal origin, 1 from environmental samples, and 1 from animal samples. The most *qnr*-positive prevalent serovar was *S*. Typhimurium followed by *S*. Saintpaul and *S*. Livingstone. None of the isolates presented the *qnr*A or *qnr*C genes.

A total of 23 isolates showed the *aac*(6′)-Ib gene, which is prevalent in *S*. Typhimurium (14/23). The most prevalent source of isolation was human (10/23), followed by foodborne (7/23), animal (3/23), and amibental (3/23). Thirteen isolates *aac*(6′)-Ib positive were resistant to all tested quinolones.

Three *qnr*-positive isolates presented the *aac*(6′)-Ib gene in association: two *S*. Typhimurium and one *S*. Saintpaul. These two *Salmonella* ser. Typhimurium were resistant to all tested quinolones/fluoroquinolones in the broth microdilution assay at the highest concentration.

Sixty-seven isolates showed the presence of integrase gene within 600 to 1000 bp variable region range and were mainly identified in human samples (38/67) followed by food samples (15/67), ambiental (8/67), and animal (6/67). The *S*. Typhimurium serovar was the most frequent (39/67) isolate with the conserved region of class 1 integron and variable regions between ±900 and >1000 bp. Nine isolates of serovar *S*. Typhimurium carrying the *aac(6*′*)-Ib* gene were positive for the integron with ±900 bp; two of these were also positive for *qnr*.

**Figure 1:**
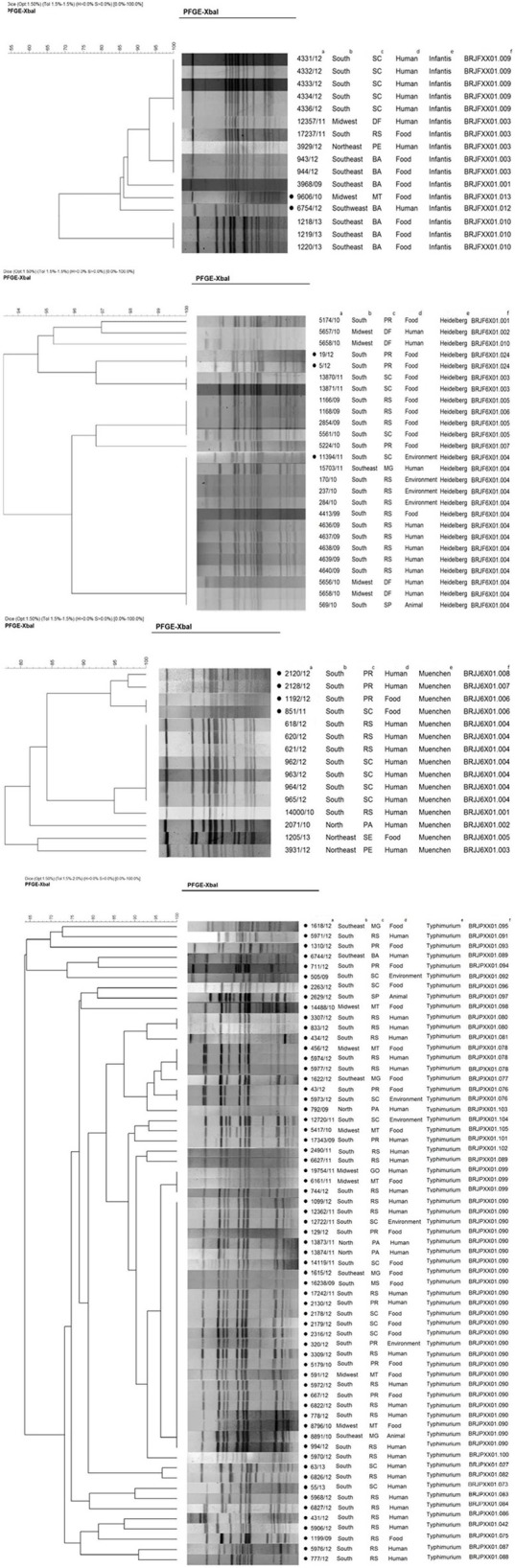
**Distribution of phylogenetic groups of *Salmonella* ser**. Infantis, Heidelberg, Muenchen, and Typhimurium according to PFGE. Isolates with resistance to quinolones; ^a^ID of IOC/year of isolation; ^b^region; ^c^state; ^d^source of isolation; ^e^serovar; ^f^pulsotype.

Figure 1 shows the clonal profile comparison between the quinolones/fluoroquinolones resistant strains and other sensitive strains.

The genetic similarity among isolates with resistance to quinolones was approximated 84% in *S*. Infantis, 92% in *S*. Heidelberg, 88% in *S*. Muenchen, and 63% in *S*. Typhimurium despite their isolation in different periods, regions, and sources.

Thirty-three distinct pulsotypes were identified among strains with low percentage genetic similarity in serovar *S*. Typhimurium (≤65%), representing the highest diversity among resistant clones.

The Table 2 presents resistance profiles obtained with the microdilution test, detection of PMQR, size of variable integron region, and the pulsotype identified by the pfge technique.

**Table 2:** **Resistance profile and resistance genes in isolates evaluated by PFGE**.

**Serovar**	**Source**	**IOC ID/Year^d^**	**Pulsotype**	**PMQR^c^**	**Integron (bp)**	**Resistance profile (MIC^b^)**
Infantis	F	6754/12	BRJFXX01.13	*qnr*D	–	CIP NAL ENO^a^
Infantis	H	9606/10	BRJFXX01.012	–	–	NAL
Heidelberg	F	19/12	BRJF0X01.024	–	700	CIP NAL ENO
Heidelberg	F	5/12	BRJF0X01.024	–	–	CIP NAL ENO
Heidelberg	H	11394/11	BRJF0X01.004	–	900	CIP NAL ENO
Muenchen	H	2120/12	BRJJ6X01.008	*qnr*S	–	NAL ENO
Muenchen	H	2128/12	BRJJ6X01.007	*qnr*S	–	NAL ENO
Muenchen	F	1192/12	BRJJ6X01.006	*aac*(6′)-Ib	600	NAL ENO
Muenchen	F	851/11	BRJJ6X01.006	-	700	CIP NAL ENO OFL
Typhimurium	F	1618/12	BRJPXX01.095	*aac*(6′)-Ib	–	CIP NAL ENO LVX OFL
Typhimurium	H	5971/12	BRJPXX01.091	*aac*(6′)-Ib	1000	NAL
Typhimurium	F	1310/12	BRJPXX01.093	*qnr*S	–	NAL ENO
Typhimurium	H	6744/12	BRJPXX01.089	*qnr*B	1000	NAL ENO
Typhimurium	F	711/12	BRJPXX01.094	*qnr*D	–	NAL
Typhimurium	E	505/09	BRJPXX01.092	–	900	CIP NAL ENO
Typhimurium	F	2263/12	BRJPXX01.096	–	–	CIP NAL ENO LVX OFL
Typhimurium	A	2629/12	BRJPXX01.097	*aac*(6′)-Ib	900	CIP NAL ENO LVX OFL
Typhimurium	F	14488/10	BRJPXX01.098	–	900	CIP NAL ENO
Typhimurium	H	3307/12	BRJPXX01.080	–	1000	CIP NAL ENO
Typhimurium	H	833/12	BRJPXX01.080	–	–	CIP NAL ENO
Typhimurium	H	434/12	BRJPXX01.081	–	–	CIP NAL ENO
Typhimurium	F	456/12	BRJPXX01.078	–	1000	NAL
Typhimurium	H	5974/12	BRJPXX01.078	–	1000	NAL OFL
Typhimurium	H	5977/12	BRJPXX01.078	–	900	CIP NAL ENO OFL
Typhimurium	F	1622/12	BRJPXX01.077	–	–	NAL ENO
Typhimurium	F	43/12	BRJPXX01.076	–	–	NAL
Typhimurium	E	5973/12	BRJPXX01.076	–	1000	CIP NAL ENO
Typhimurium	H	792/09	BRJPXX01.103	–	900	CIP NAL
Typhimurium	E	12720/11	BRJPXX01.104	–	900	CIP NAL ENO LVX OFL
Typhimurium	F	5417/10	BRJPXX01.105	–	900	CIP NAL ENO LVX OFL
Typhimurium	H	17343/09	BRJPXX01.101	–	1000	CIP NAL ENO
Typhimurium	H	2490/11	BRJPXX01.102	–	900	CIP NAL ENO LVX OFL
Typhimurium	H	6627/11	BRJPXX01.089	–	900	CIP NAL ENO LVX OFL
Typhimurium	H	19754/11	BRJPXX01.099	–	–	CIP NAL ENO LVX OFL
Typhimurium	F	6161/11	BRJPXX01.099	–	–	CIP NAL ENO LVX OFL
Typhimurium	H	744/12	BRJPXX01.099	–	900	CIP NAL ENO LVX OFL
Typhimurium	H	1099/12	BRJPXX01.090	–	1000	CIP NAL ENO LVX OFL
Typhimurium	H	12362/11	BRJPXX01.090	–	900	CIP NAL ENO LVX OFL
Typhimurium	E	12722/11	BRJPXX01.090	–	–	CIP NAL ENO LVX OFL
Typhimurium	F	129/12	BRJPXX01.090	*aac*(6′)-Ib	–	CIP NAL ENO LVX OFL
Typhimurium	H	13873/11	BRJPXX01.090	–	900	CIP NAL ENO LVX OFL
Typhimurium	H	13874/11	BRJPXX01.090	–	900	CIP NAL ENO LVX OFL
Typhimurium	F	14119/11	BRJPXX01.090	–	900	CIP NAL ENO LVX OFL
Typhimurium	F	1615/12	BRJPXX01.090	–	–	CIP NAL ENO LVX OFL
Typhimurium	F	16238/09	BRJPXX01.090	–	900	CIP NAL ENO LVX OFL
Typhimurium	H	17242/11	BRJPXX01.090	–	900	CIP NAL ENO LVX OFL
Typhimurium	H	2130/12	BRJPXX01.090	*aac*(6′)-Ib	–	CIP NAL ENO LVX OFL
Typhimurium	F	2178/12	BRJPXX01.090	*aac*(6′)-Ib	–	CIP NAL ENO LVX OFL
Typhimurium	F	2179/12	BRJPXX01.090	*aac*(6′)-Ib	–	CIP NAL ENO LVX OFL
Typhimurium	F	2316/12	BRJPXX01.090	–	900	CIP NAL ENO LVX OFL
Typhimurium	E	320/12	BRJPXX01.090	*aac*(6′)-Ib	900	CIP NAL ENO LVX OFL
Typhimurium	H	3309/12	BRJPXX01.090	*qnr*B*/aac*(6′)-Ib	900	CIP NAL ENO
Typhimurium	F	5179/10	BRJPXX01.090	–	–	CIP NAL ENO
Typhimurium	F	591/12	BRJPXX01.090	–	900	NAL
Typhimurium	H	5972/12	BRJPXX01.090	*aac*(6′)-Ib	900	CIP NAL ENO LVX OFL
Typhimurium	F	667/12	BRJPXX01.090	–	–	CIP NAL ENO
Typhimurium	H	6822/12	BRJPXX01.090	–	900	CIP NAL ENO LVX OFL
Typhimurium	H	778/12	BRJPXX01.090	*qnr*B	–	CIP NAL ENO LVX OFL
Typhimurium	F	8796/10	BRJPXX01.090	–	900	CIP NAL ENO LVX OFL
Typhimurium	A	8891/10	BRJPXX01.090	–	900	CIP NAL ENO LVX OFL
Typhimurium	H	994/12	BRJPXX01.090	–	900	CIP NAL ENO OFL
Typhimurium	H	5970/12	BRJPXX01.100	*aac*(6′)-Ib	–	CIP NAL ENO LVX OFL
Typhimurium	H	63/13	BRJPXX01.027	–	1000	CIP NAL ENO
Typhimurium	H	6826/12	BRJPXX01.082	–	1000	NAL
Typhimurium	H	55/13	BRJPXX01.073	*qnr*D	–	CIP NAL ENO LVX OFL
Typhimurium	H	5968/12	BRJPXX01.083	–	1000	NAL
Typhimurium	H	6827/12	BRJPXX01.084	–	1000	NAL
Typhimurium	H	431/12	BRJPXX01.086	–	–	–
Typhimurium	H	5906/12	BRJPXX01.042	–	–	NAL
Typhimurium	F	1199/09	BRJPXX01.075	–	600	CIP NAL ENO
Typhimurium	H	5976/12	BRJPXX01.087	*aac*(6′)-Ib	700	CIP NAL ENO LVX OFL
Typhimurium	H	777/12	BRJPXX01.088	*qnr*B*/aac*(6′)-Ib	–	CIP NAL ENO LVX OFL

a *CIP, Ciprofloxacin; ENO, Enrofloxacin; NAL, Nalidixic Ácid; LVX, Levofloxacina; OFL, Ofloxacin*;

b *MIC, Minimum Inhibitory Concentration*;

c *PMQR, Plasmid-Mediated Quinolone Resistance*;

d *IOC ID/Year, Institut Oswaldo Cruz Identification by Year*;

*^e^F, Food; A, Animal; E, Environment; H, Human*.

## Discussion

The variation in resistance to the different tested quinolones can be explained by the mechanism of resistance when the resistance level depends on the affected target enzyme, the number of accumulated mutations, and presence of PMQRs. Furthermore, there is a relationship between the level of specific resistance and potency of each drug, especially in the newest quinolones (Sanders, 2001; Ruiz et al., 2012).

Chong et al. (2010) reported that an increased resistance to fluoroquinolones based on the acquisition of *qnr* genes could be related with reduction in the clinical efficacy “of this class” of antimicrobial. “However, Jacoby et al. (2009)” argue that the genes involved in plasmid-level resistance to fluoroquinolones are still poorly understood when compared to other resistance mechanisms.

A high prevalence of isolates carrying PRQM genes is reported in the present study (27%, 35/129). The most prevalent serovar associated with the presence of PMQR genes was *Salmonella* ser. Typhimurium (18/35). A high level of detection of *S*. Typhimurium was expected because this serovar is directly related to detected genotypic and phenotypic profiles of antimicrobial resistance (Herrero et al., 2008; Kingsley et al., 2009). The presence of PMQR genes is related “to decreased susceptibility to fluoroquinolones,” accelerating the selection of fluoroquinolone-resistant mutants (Rodríguez-Martínez et al., 2011).

Three isolates presented association between the *qnr* and *aac*(6′)-Ib genes. A similar association has been reported by Park et al. (2006) in the United States, Xiong et al. (2011) while investigating the *aac*(6′)-Ib and *qnr* genes in *Enterobacter cloacae* “in China, and” Kim et al. (2013) in enterobacteria isolated from clinical samples in Korea. Not sequencing the *aac*(6′)-Ib gene to determine the cr variant was one limitation in the present study.

Regardless that some authors recognize the location of the *aac*(6′)-Ib gene mostly in class 1 integrons, our results show the absence of this gene in all analyzed strains [12 *aac*(6′)-Ib positive isolates without the integron region] (Rodríguez-Martínez et al., 2011; Kim et al., 2013).

The Enteritidis serovar was not assessed by PFGE because, according to the literature, these isolates have low clonal diversity (Spiliopoulou et al., 2007).

Six distinct pulsotypes were detected in *S*. Infantis serovar isolates. Those with resistance to quinolones are placed in two separate pulsotypes (BRJFXX01.13 and BRJFXX01.12) with a genetic similarity of ~85%. The quinolone resistance isolates were obtained from different sources, regions, and periods, and the resistance to quinolones showed variations. The 6754/12 isolate showed resistance to ciprofloxacin, nalidixic acid, and enrofloxacin and carried the *qnr*D gene; the 9606/10 isolate showed resistance to nalidixic acid only and did not carry resistant genes.

The *S*. Heidelberg serovar presented eight distinct pulsotypes. The resistant isolates showed a clonal ratio of 100% similarity between the isolates 5/12 and 19/12, and ~94% between them and isolate 11394/11. The 11394/11 isolate (environmental source from the Southern region) was detected in the BRJF6X01.004 pulsotype (pulsotype with 13 susceptible isolates). Isolates 5/12 and 19/12, within the same pulsotype, were foodborne and originated in the Southern region.

Three pulsotypes were identified in serovar Muenchen isolates with quinolone resistance profiles (pulsotypes BRJJ6X01008, BRJJ6X01007, and BRJJ6X01006), showing 88% of genetic similarity. Among the isolates resistant, the isolates of human origin presenting a profile similarity of ~97%. The isolates of foodborne origin presenting the same origin clonal being from different periods and states. The 2128/12, 2120/12, and 1192/12 isolates show similar quinolone resistance profiles. However, isolate 851/11 show a resistance profile to ciprofloxacin and ofloxacin. The 2120/12 and 2128/12 isolates show similar resistance profile and are carriers of the *qnr*S gene.

The detection of 33 different pulsotypes of *S*. Typhimurium indicates that different clones with resistance to quinolones are circulating in Brazil. The most prevalent pulsotype (BRJPXX01.090) is mainly represented in samples from the Southern region and are related to food and human sources. Most isolates of this pulsotype show the same resistance profile to quinolones/fluoroquinolones (except isolates 3309/11, 5179/10, 591/12, and 667/12) demonstrating a resistance profile to all tested quinolones/fluoroquinolones. Out of the 25 isolates showing resistance to quinolones, 4 did not carry resistance genes. Twelve isolates show an integron with a variable region of 900 bp and one with >1000 bp. One isolate shows the *qnr*B gene and, four show the *aac*(6′)-Ib gene. Two isolates show the 900 bp integron and the *aac*(6′)-Ib gene; one isolate shows the 900 bp integron and the *qnr*B, *acc*(6′)-Ib gene.

The profiles identified in the PFGE analysis show relatively high diversity in all serovars and, indicate that cases of resistance to quinolones are probably sporadic. This interpretation is in accordance with other results reported in the literature (Feasey et al., 2012).

## Author contributions

All authors listed, have made substantial, direct and intellectual contribution to the work, and approved it for publication.

### Conflict of interest statement

The authors declare that the research was conducted in the absence of any commercial or financial relationships that could be construed as a potential conflict of interest.
